# Laparoscopic ultrasound-guided superselective portal vein injection combined with real-time indocyanine green fluorescence imaging and navigation for accurate resection of localized intrahepatic bile duct dilatation: a case report

**DOI:** 10.1186/s12893-021-01325-w

**Published:** 2021-08-17

**Authors:** Ming-chun Lai, Lei Geng, Shu-sen Zheng, Jun-fang Deng

**Affiliations:** Division of Hepatobiliary and Pancreatic Surgery, Department of Surgery, The First Affiliated Hospital, Zhejiang University School of Medicine, 79 Qingchun Road, Hangzhou, 310003 People’s Republic of China

**Keywords:** Localized bile duct dilatation, Indocyanine green fluorescence imaging and navigation, Laparoscopic surgery

## Abstract

**Background:**

Primary intrahepatic bile duct dilatation can be very harmful to patients although it belongs to benign biliary disease. It can occur in any part of the liver, intraoperative laparoscopic ultrasound (LUS) guidance combine with real-time indocyanine green (ICG) fluorescence navigation are the means of choice for accurate surgical resection.

**Case presentation:**

Herein we reported a 43-year-old female patient presented with repeated right upper abdominal pain and distension for 3 years and aggravated for half a year, without fever and jaundice. A diagnosis of localized bile duct dilatation with lithiasis in segment 4 (S4) was made on the basis of preoperative imaging. Correspondingly, we selected to perform a laparoscopic surgery with LUS guided real time ICG fluorescence imaging (ICG-FI) and navigation to make the operation more simply and accurately, as well as to retain normal tissues in a certain extent. Laparoscopic resection of S4b and partial S4a was successfully performed, without any complications.

**Conclusion:**

Laparoscopic anatomical surgery for intrahepatic bile duct dilatation is a technically challenging operation. The combined use of preoperative three-dimensional computerized tomography (CT) planning, intraoperative LUS guided super-selection, ICG hepatic segment staining and real-time fluorescence navigation could help surgeons accurately complete the segmentectomy or subsegmentectomy with minimized trauma and maximized liver tissue preservation.

**Supplementary Information:**

The online version contains supplementary material available at 10.1186/s12893-021-01325-w.

## Background

Primary intrahepatic bile duct dilatation is a common biliary tract disease in China, and its clinical manifestations are mainly related to the onset of cholangitis, stone formation, liver abscess and so on. Repeated bacterial infection and mechanical stimulation of hepatolithiasis will lead to local bile duct dilatation, cholangitis, mucosal dysplasia and even progression to cholangiocarcinoma, which in the range of 7 to 14% [1]. Resection of the liver segment where the dilated bile duct located could ease the symptoms and complications and reduce the risk of recurrence and cholangiocarcinoma. Laparoscopic partial hepatectomy has gradually become a routine surgical method in hepatobiliary surgery in recent years, which has obvious advantages over traditional open surgery, with less bleeding, lower incidence of complications and shorter hospital stay [2].

Recently, in vivo ultrasonic and fluorescence imaging techniques to identify biological structures during laparoscopic surgical treatment have been developed greatly. Among which indocyanine green (ICG) has gained widespread acceptance in laparoscopic hepatectomy (LH) [3]. With the retention and accumulation effect of ICG in lesions, real-time fluorescence imaging can yield visualization of the hepatic parenchyma and biliary tract during LH surgery [4, 5]. Thus, the ICG fluorescence imaging (ICG-FI) technique might be able to clearly identify hepatic segmental boundaries and the location of tumors, helping operators to timely adjust the surgical approach and accurately dissect the hepatic segments, avoid tumor residue and reduce vessel damage. ICG-FI has shown its unique value in anatomical LH for hepatic malignancies, but it is rarely used in the treatment of the intrahepatic bile duct dilatation or hepatolithiasis, which is worthy of further exploration and application. Herein we report a case of localized S4 intrahepatic bile duct dilatation treated successfully with accurate surgical resection by laparoscopic ultrasound-guided superselective portal vein injection combined with real-time ICG-FI and navigation.

## Case report

**Fig. 1:**
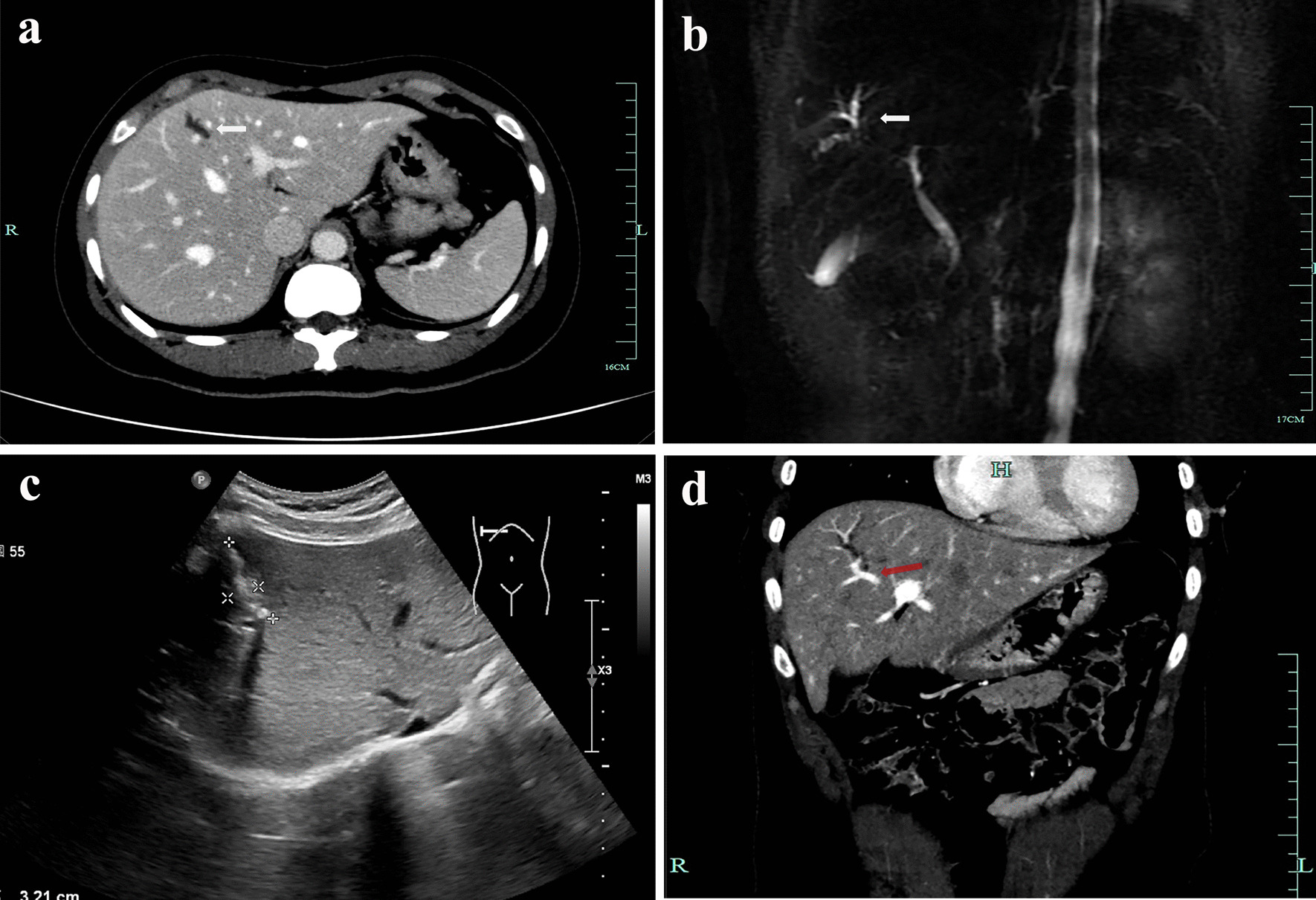
**a** Preoperative computed tomography imaging revealed localized intrahepatic bile duct dilatation in the superior region of hepatic S4 (white arrow); **b** Magnetic resonance cholangiopancreatography showed localized intrahepatic bile duct dilatation in the region of hepatic S4 (white arrow); **c** Ultrasound examination showed localized intrahepatic bile duct dilatation with cholangiolithiasis in the region of hepatic S4 (specific tag); **d** Computed tomographic angiography indicated the portal vein branch of the target hepatic segment (red arrow)

A 43-year-old female patient with localized intrahepatic bile duct dilatation was found by physical examination for 20 years. For recent 3 years, the patient felt repeated right upper abdominal pain and distension, and aggravated for half a year, without fever and jaundice. She was admitted to our hospital for further examination and treatment. The patient's physical examination and hematological tests, including tumor markers Ca-199 were unremarkable. Enhanced CT and magnetic resonance cholangiopancreatography (MRCP) suggested that the intrahepatic bile duct dilatation in segment 4(S4) with an extent of 2.5*3.0 cm (Fig. 1a, b). Hepatobiliary ultrasound revealed localized dilatation of bile duct with local acoustic enhancement in S4 of the liver (Fig. 1c). On the basis of these findings, a diagnosis of S4 localized bile duct dilatation with lithiasis was made. Given the recurrent and persistent symptoms, the patient opted for surgical resection of biliary dilatation. Correspondingly, we selected to perform a combined resection of segment 4b and part of 4a with the guidance of subsegmental ICG staining, so as to facilitate the laparoscopic operation more simply and accurately, as well as to retain normal tissues in a certain extent.

### Surgical procedure

**Fig. 2:**
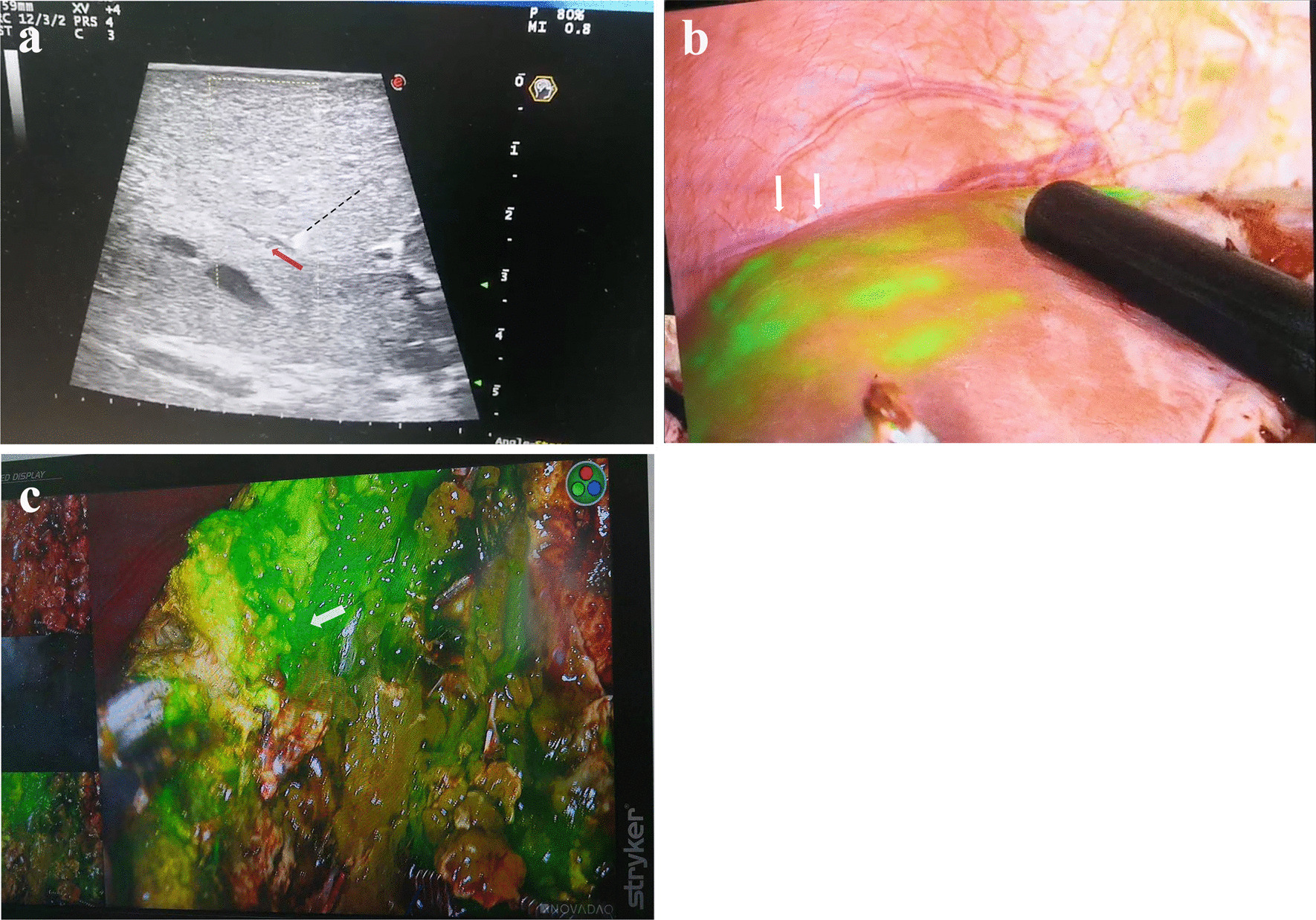
**a** Portal vein branch of the target hepatic segment was super-selected and punctured with a 18G needle by intraoperative laparoscopic ultrasound guidance (black dotted line), then an injection of 0.05 mg indocyanine green was performed gently (0.025 mg/ml, red arrow); **b** Visualization of the lesion area by fluorescence imaging after the intraoperative portal vein injection of indocyanine green (white arrow); **c** Real-time indocyanine green fluorescence imaging (ICG-FI) enabled the visualization of overall structure of the lesion and intrahepatic surgical boundary during operation (white arrow)

**Fig. 3:**
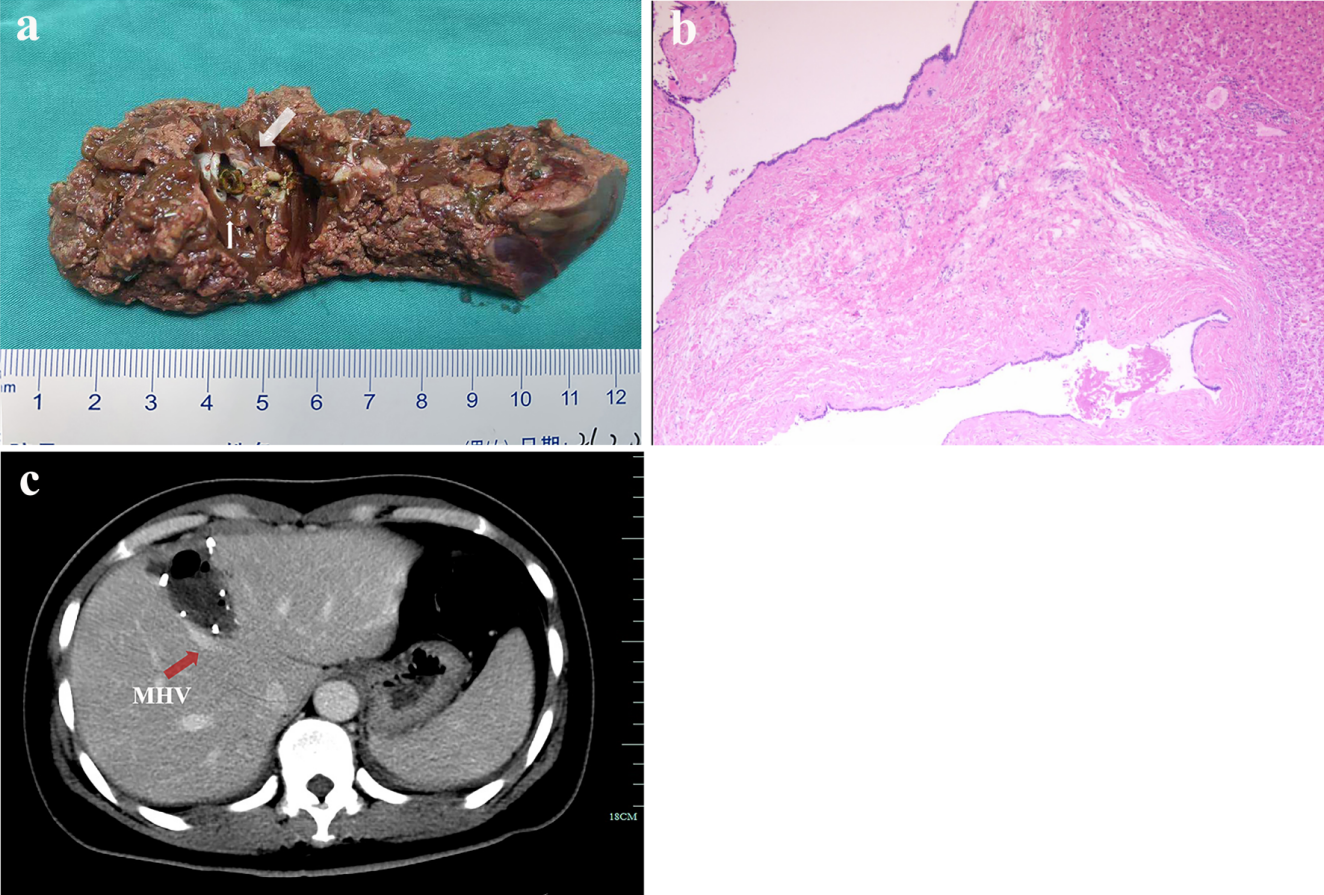
**a** Localized bile duct dilatation with lithiasis was confirmed after hepatic resection with the aid of indocyanine green fluorescence imaging and navigation (white arrow); **b** Pathology demonstrated bile duct dilatation with inflammatory cell infiltration; **c** Postoperative enhanced CT confirmed the integrity of the resection and the location of middle hepatic vein (MHV, red arrow)

Intraoperatively, after mobilization of the liver, relationships of the pathological segment with the hepatic vessels were confirmed by LUS, which was consistent with the preoperative CT imaging (Fig. 1d). ICG fluorescence positive staining method was performed to define the exact anatomical location of the lesion. Portal vein branch of the target hepatic segment was super-selected and visualized longitudinally, followed by punctured with a 18G needle by LUS guidance, then an injection of 0.05 mg ICG was performed gently (Fig. 2a, Additional file 1). The specific configuration method of dilution was as follows: 25 mg ICG was dissolved in 10 ml sterilized water, and then taken 1 ml of which into 100 ml saline. Significantly, special attention must be paid to avoid the dye entering to other hepatic lobes. After injection of ICG, fluorescence imaging could highlight the segmental resection line, the non-fluorescent hepatic tissues were also identified (Fig. 2b). Fusion fluorescence images were obtained using the PINPOINT imaging system (Stryker, Kalamazoo, USA). The imaging was switched to the fluorescent mode when predicting that the surgical boundary of the lesion would be exposed, then enabled visualization of the lesion as a clearly distinguished region of green fluorescence during operation (Fig. 2c). Since the lesion was located between S4a and S4b, indicating the need for partial resection of the segment 4 rather than removing the whole S4. There were no complications during the procedure, with an operation time of 90 min and a bleeding volume of 100 ml, and the size of the resected hepatic segment was 9*4 cm (Fig. 3a). Final pathology revealed presence of chronic and fibrosing inflammation with dilatation and cholelithiasis in local bile duct (Fig. 3b), and post-operative enhanced CT confirmed the integrity of the resection (Fig. 3c). The patient was recovered soon, then discharged 6 days after operation, and she has not experienced similar symptoms as before during the first three month of follow-up.

## Discussion

This clinical case report documented that application of the newly developed real-time ICG-FI display system during laparoscopic hepatobiliary surgery for intrahepatic bile duct dilatation was safe and feasible. It provided a significantly higher recognition of both surgical boundary and vessels, assisting in safe, efficient and accurate dissection of specified hepatic segment.

The satisfactory therapeutic effect for intrahepatic biliary dilatation should achieved the purpose of eliminating stones and resecting the liver segment involved by dysfunction [1]. Long-term outcome of minimally invasive hepatectomy is comparable to that of the open approach, with verified benefits in terms of less surgical trauma and bleeding with the procedure, and less clinical complications and shorter length of hospitalization. During a precision LH, intraoperative LUS assessment is indispensable for confirmation of the location and extent of the lesions, as well as distribution of the hepatic segment glisson pedicle, so as to avoid the injury of vessels [6]. In addition, intraoperative ICG-FI technology combines the metabolism and functional insight into morphological imaging, providing a simple and effective navigational tool for surgeons to judge the stereoscopic staining more intuitively and accurately during operation [7, 8]. With the help of intraoperative navigation technique, the operator could dynamically observe the fluorescent images on the monitor and adjust the boundaries, finally achieve anatomical, functional and radical hepatectomy.

Compared with conventional methods, LUS combined with fluorescence imaging enable the cross-sectional boundaries more conducive in segmental LH, which solves the disadvantage of lack of tactile feedback in laparoscopic surgery [9, 10]. As we know, it can be difficult to accurately puncture the branches of the portal vein of the target liver segment during a laparoscopic approach. Therefore, the surgeon must make sure that he has the right mix of capabilities to combine use of fluorescence technique, ultrasound examination, puncture technique and surgical anatomy itself, which puts forward higher requirements for minimally invasive surgeons in the future.

It is known that due to the relatively large number of hepatic communicating branches, it is necessary to accurately evaluate the vessels supply of the specified liver segments by combining preoperative CT and intraoperative LUS in order to improve the success rate of ICG staining [11]. Meanwhile, after confirming that the puncture entered the target portal vein by LUS, ICG should be injected fairly slow, so as to prevent the fluorescent dye from entering other hepatic tissues. Currently, there is no accepted standard regarding dosage or concentration of ICG injection. In general, the amount of ICG should be reduced if possible to avoid excessive fluorescence staining that makes the boundary between fluorescent and non-fluorescent area become unclear [5]. In addition, it should be also avoided ICG injecting into the hepatic vein branch and then entering through the systemic circulation, even a small amount of dye, which may stain whole liver and result in staining failure [4, 5, 12]. Even so, at some point, there is often some inevitably fluorescent noise in the hepatic tissues. Could it be because there are some connecting vessels between the portal vein and the hepatic vein, which enable some ICG to skip the metabolic process, then appear in systemic circulation [3]. Another thing to be note is that as well as the appropriate concentration of ICG, the camera could be switched to fluorescence mode during the injection, and the process terminated immediately when the target tissue has enhanced dye up-taking. The anatomy of the segment 4 is highly individualized. Sometimes there would be several branched roots, it is difficult for any method to successfully complete the staining for multiple subsegments. In our experience, the positive staining method should be used for segmental or subsegmental staining. After successful puncture and injection of appropriate concentration and dose of ICG, the staining of the target hepatic segment will persist almost eight hours, and the range of staining will not change throughout the surgery. In the case of multiple hepatic segments or hemihepatic resection, the increase of target Glissonean branches will further aggravate the difficulty of puncture, and the ICG dosage is hard to grasp in positive staining. In addition, there will be uneven blood flow due to liver rotation and compression, resulting in uneven dyeing, so negative staining should be selected at this time [13]. Additionally, ICG is filtered by hepatocytes and excreted in the bile, so utilizing real-time fluorescence cholangiography in patients with complicated or aberrant biliary anatomical structure can further improve the surgery efficacy, accuracy and safety of the operation [1, 14]. This is an issue which also worth to be studied.

 To sum up, laparoscopic anatomical surgery for intrahepatic bile duct dilatation is a technically challenging operation. The combined use of preoperative three-dimensional CT planning, intraoperative laparoscopic ultrasound guided super-selection, ICG hepatic segment staining and real-time fluorescence navigation could help surgeons accurately complete the segmentectomy or subsegmentectomy with minimized trauma and maximized liver tissue preservation.

## Supplementary Information

**Additional file 1**. Injection of indocyanine green to target hepatic segment pedicle.

## Data Availability

All clinical data and images adopted in this article are contained in the medical files of the First Affiliated Hospital, Zhejiang University School of Medicine.

## Abbreviations

ICG: Indocyanine green
ICG-FI: ICG fluorescence imaging
CT: Computed tomography
MRCP: Magnetic resonance cholangiopancreatography
LUS: Laparoscopic ultrasound
LH: Laparoscopic hepatectomy
Segment 4: S4
